# Impact of the 21-gene expression assay on treatment decisions and clinical outcomes in breast cancer with one to three positive lymph nodes

**DOI:** 10.3389/fendo.2023.1103949

**Published:** 2023-02-16

**Authors:** Guan-Qiao Li, Shang-Jin Xie, San-Gang Wu, Zhen-Yu He

**Affiliations:** ^1^ Department of Breast Surgery, Hainan General Hospital (Hainan Affiliated Hospital of Hainan Medical University), Haikou, China; ^2^ Department of General Surgery, Xiang’an Hospital of Xiamen University, Xiamen, China; ^3^ Department of Radiation Oncology, Xiamen Cancer Center, Xiamen Key Laboratory of Radiation Oncology, The First Affiliated Hospital of Xiamen University, School of Medicine, Xiamen University, Xiamen, China; ^4^ Department of Radiation Oncology, Sun Yat-sen University Cancer Center, State Key Laboratory of Oncology in South China, Collaborative Innovation Center of Cancer Medicine, Guangzhou, China

**Keywords:** breast cancer, lymph nodes, oncotype, survival, chemotherapy

## Abstract

**Background:**

To assess the practice patterns of the recurrence score (RS) based on the 21-gene expression assay on adjuvant chemotherapy recommendations and survival outcomes in estrogen receptor-positive (ER+)/HER2- breast cancer (BC) with one to three positive lymph nodes (N1).

**Methods:**

We included patients with T1-2N1M0 and ER+/HER2- BC diagnosed between 2010 and 2015 in the Surveillance, Epidemiology, and End Results Oncotype DX Database. Breast cancer-specific survival (BCSS) and overall survival (OS) were assessed.

**Results:**

We included 35,137 patients in this study. There were 21.2% of patients who had RS testing in 2010, which was significantly increased to 36.8% in 2015 (P < 0.001). Performance of the 21-gene testing was associated with older age, lower tumor grade, T1 stage, lower number of positive lymph nodes, and progesterone receptor-positive disease (all P < 0.05). In those without 21-gene testing, age was the main factor significantly related to the receipt of chemotherapy, whereas RS was the main factor significantly related to chemotherapy receipt in those with 21-gene testing. The probability of chemotherapy receipt in those without 21-gene testing was 64.1% and was decreased to 30.8% in those with 21-gene testing. On multivariate prognostic analysis, the performance of 21-gene testing was associated with better BCSS (P < 0.001) and OS (P < 0.001) compared with those without 21-gene testing. Similar results were found after propensity score matching.

**Conclusions:**

The 21-gene expression assay is frequently and increasingly used for chemotherapy decision-making in ER+/HER2- BC with N1 disease. Performance of the 21-gene testing is associated with improved survival outcomes. Our study supports the routine use of 21-gene testing in the clinical practice of this population.

## Background

Breast cancer (BC) is the most common malignancy diagnosed among women, with approximately 2.2 million new cases annually (1). With the improvement of diagnosis and screening technology, there were 64.2% of the patients who had a node-negative (N0) disease at BC diagnosis, but 35.8% of the patients still had a node-positive (N+) disease. Among those with N+ diseases, 84.5%, 10.4%, and 5.1% had N1, N2, and N3 diseases, respectively (2). More than 70% of BC patients are hormone receptor (HR)-positive (2). For HR-positive early BC with metastasis (N1) of one to three lymph nodes (LNs), endocrine therapy after surgery is the standard treatment strategy, but the role of chemotherapy in this population remains controversial (3, 4). However, approximately 15% of patients with N+ and HR+/HER2- BC receiving endocrine therapy develop tumor recurrence within 5 years of initiating treatment, indicating a requirement for developing novel treatment strategies in this patient subset (5). With increasing knowledge regarding the molecular heterogeneity of BC, the recurrence score (RS) based on the 21-gene expression assay has revolutionized the chemotherapy decision-making in N0 and estrogen receptor-positive (ER+) BC patients (6). The value of RS in N0 and ER+/HER2- early-stage BC has been validated for the first time in a landmark study, indicating that those with RS ≥31 would benefit from additional adjuvant chemotherapy but those with RS <18 would not (7). However, the RS testing in the N+ BC patients has been limited but is steadily increasing, particularly following the recent findings from the data of RxPONDER (8).

For patients with N1 BC, whether treatment decisions can be made according to the results of the 21-gene RS, the current treatment guidelines from the American Society of Clinical Oncology (ASCO) and the National Comprehensive Cancer Network (NCCN) have contradictory opinions (4, 9). However, several previous studies have found that the 21-gene RS could also predict the survival of N1 patients and have an impact on treatment decisions (8, 10–13). In Canada, the 21-gene RS has been publicly funded for patients with N0 and ER+/HER2- BC for several years, whereas the coverage of RS testing for N+ patients has been very limited (14). Given emerging data on the role of RS and the recent discrepant treatment in these patients, this study aimed to clarify the practice patterns and the potential benefit of 21-gene testing in BC patients with ER+/HER2− and one to three LNs.

## Materials and methods

### Patients

We identified patients who were diagnosed with BC from the Surveillance, Epidemiology, and End Results (SEER) Oncotype DX Database (2004–2016) with or without SES/Rurality (15), which includes 21-gene RS data for invasive BC patients diagnosed from 2004 to 2015. Patients included in this database had follow-up through the end of 2016. We limited this analysis to pathologic stage T1-2N1M0 and ER+/HER2- BC with the availability of RS diagnosed between 2010 and 2015 because the data regarding HER2 status were only included from the year 2010. We excluded those with missing surgery and radiotherapy information and those with missing tumor grade, progesterone receptor (PR) status, and insurance status. This study did not require institutional review board approval because the data in the SEER program were deidentified.

### Variables

The following variables were included in the analysis: age, race, tumor grade, histology, tumor (T) stage, PR status, number of positive LNs, surgical procedure, and the receipt of radiotherapy, chemotherapy, and RS testing. Patients who underwent 21-gene testing were further stratified into low (RS <18), intermediate (RS 18–30), and high (RS >30) groups (6). We used the above pre-TAILORx RS categories because the patients included in this study were treated before the publication of the TAILORx study (16). The primary outcome measures were breast cancer-specific survival (BCSS) and overall survival (OS).

### Statistical analysis

Patients’ baseline characteristics were compared according to the receipt of the 21-gene testing using chi-square tests. Multiple logistic regression models were used to determine the predictive factors related to the use of RS testing and chemotherapy. A multicollinearity test was used to assess the data collected. The Kaplan–Meier method was used to assess differences in BCSS and OS and compared by the log-rank test. Multivariable Cox proportional hazards models were performed to determine independent prognostic factors impacting BCSS and OS. A 1:1 propensity score matching (PSM) was used to balance the potential confounders. Statistical analyses were conducted using the SPSS version 25.0. A P value less than 0.05 was considered statistically significant.

## Results

### Patients’ baseline characteristics

**Figure 1:**
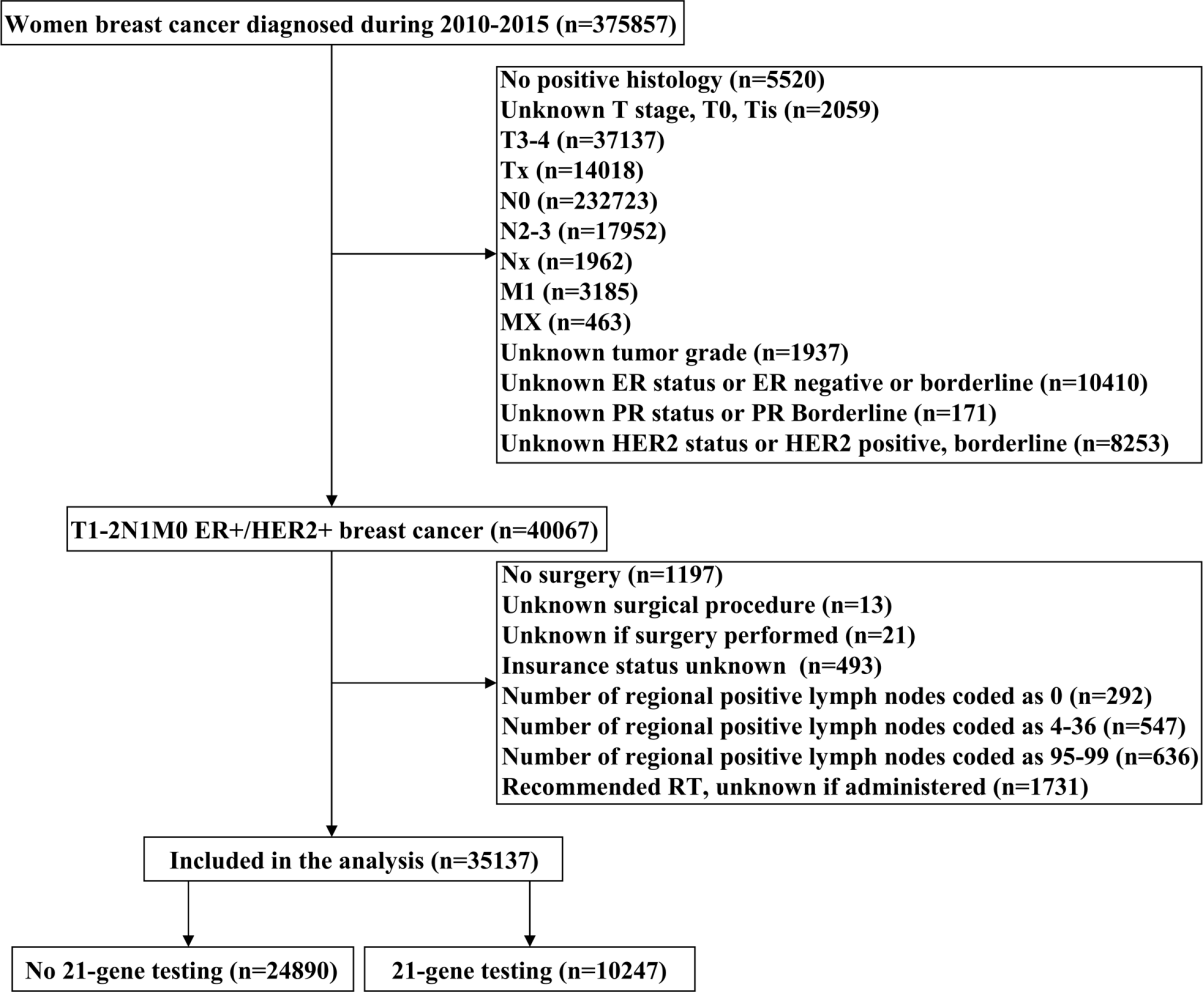
The patient selection flowchart of this study.

We included 35,137 patients in this study (**Figure 1** and **Table 1**). Of these patients, 69.6% (n = 24,466) were non-Hispanic white, 87.5% (n = 30,733) were invasive ductal carcinoma, and 89.5% (n = 31458) were PR positive. There were 23,222 (66.1%), 8,234 (23.4%), and 3,681 (10.5%) patients who had one-, two-, and three-LN metastases, respectively. Regarding treatment, 50.5% (n = 17,762), 55.7% (n = 19,572), and 54.4% (n = 19,118) patients received mastectomy, radiotherapy, and chemotherapy, respectively.

**Table 1:** Patient baseline characteristics before and after propensity score matching.

Variables	Before PSM				After PSM			
	n	No 21-gene testing (%)	21-gene testing(%)	P	n	No 21-gene testing	21-gene testing	P
Age (years)								
<50	8,401	6,408 (25.7)	1,993 (19.4)	<0.001	2,762	1381	1,381	1
50-64	13,952	9,413 (37.8)	4,539 (44.3)		6,116	3058	3,058	
≥65	12,784	9,069 (36.4)	3,715 (36.3)		6,878	3439	3,439	
Race/ethnicity								
Non-Hispanic white	24,466	16,946 (68.1)	7,520 (73.4)	<0.001	11,888	5944	5,944	1
Non-Hispanic Black	3,405	553 (10.3)	852 (8.3)		1,200	600	600	
Hispanic (all races)	4,094	3,109 (12.5)	985 (9.6)		1,478	739	739	
Other	3,172	2,282 (9.2)	890 (8.7)		1,190	595	595	
Histology								
Invasive ductal carcinoma	30,733	21,810 (87.6)	8,923 (87.1)	0.002	14,006	7,003	7,003	1
Invasive lobular carcinoma	3,710	2,559 (10.3)	1,151 (11.2)		1,570	785	785	
Other	694	521 (2.1)	173 (1.7)		180	90	90	
Grade								
Well differentiated	7,555	4,786 (19.2)	2,769 (27.0)	<0.001	3,868	1,934	1,934	1
Moderately differentiated	19,213	13,313 (53.5)	5,900 (57.6)		9,220	4,610	4,610	
Poorly/undifferentiated	8,369	6,791 (27.3)	1,578 (15.4)		2,668	1,334	1,334	
T stage								
T1	18,628	12,107 (48.6)	6,521 (63.6)	<0.001	9,662	4,831	4,831	1
T2	16,509	12,783 (51.4)	3,726 (36.4)		6,094	3,047	3,047	
Number of positive lymph nodes								
1	23,222	15,138 (60.8)	8,084 (78.9)	<0.001	12,240	6,120	6,120	1
2	8,234	6,538 (26.3)	1,696 (16.6)		2,786	1,393	1,393	
3	3,681	3,214 (12.9)	467 (4.6)		730	365	365	
PR status								
Negative	3,679	2,929 (11.8)	750 (7.3)	<0.001	1,086	543	543	1
Positive	31,458	21,961 (88.2)	9,497 (92.7)		14,670	7,335	7,335	
Surgery procedure								
Breast-conserving surgery	17,375	11,263 (45.3)	6,112 (59.6)	<0.001	8,744	4,372	4,372	1
Mastectomy	17,762	13,627 (54.7)	4,135 (40.4)		7,012	3,506	3,506	
Radiotherapy								
No/unknown	15,565	11,605 (46.6)	3,960 (38.6)	<0.001	7,002	3,501	3,501	1
Yes	19,572	13,285 (53.4)	6,287 (61.4)		8,754	4,377	4,377	
Chemotherapy								
No	16,019	8,931 (35.9)	7,088 (69.2)	<0.001	9,680	4,840	4,840	1
Yes	19,118	15,959 (64.1)	3,159 (30.8)		6,076	3,038	3,038	

**Figure 2:**
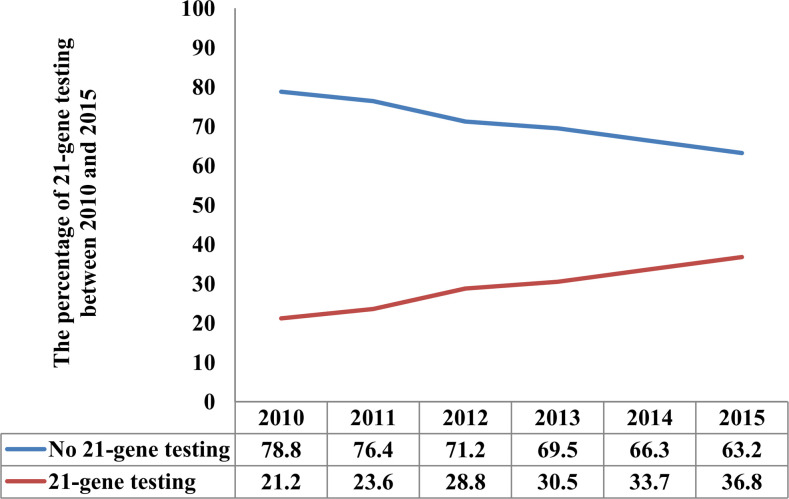
The proportion of 21-gene testing over time.

There were 29.2% (n = 10,247) of patients who had RS testing. Of these patients, the median RS was 15 (range, 0–67). In those with RS testing, there were 6,265 (61.1%), 3,405 (33.2%), and 577 (5.6%) patients who had low, intermediate, and high RSs, respectively. The proportion of RS testing over time is listed in **Figure 2**. There were 21.2% of patients who had RS testing in 2010, which was significantly increased to 36.8% in 2015 (P < 0.001). Moreover, patients aged 50–64 years; are non-Hispanic white; had a lower tumor grade, T1 stage, one positive LN, PR positive; receiving BCS (P < 0.001); and receiving RT were more likely to have RS testing (all P < 0.001) (**Table 1**). Moreover, patients receiving RS testing were less likely to receive chemotherapy compared with those that did not receive RS testing (30.8% vs. 64.1%, P < 0.001).

### Predictive factors associated with 21-gene testing

Multicollinearity was checked for all predictors by tolerance analysis. All of the predictors’ tolerance was above the cutoff of 0.10 (ranging between 0.918 and 0.980), suggesting that there was no risk of multicollinearity. The predictive factors associated with 21-gene testing were then identified using binomial logistic regression. The results showed that older age, non-Hispanic white, invasive ductal carcinoma subtype, lower tumor grade, T1 stage, lower number of positive LNs, and PR positive disease were the independent predictive factors associated with the receipt of 21-gene testing (all P < 0.05) (**Table 2**). Those with three positive LNs had the lowest chance of RS testing, with an odds ratio (OR) of 0.307 compared with those with one positive LN (95% CI 0.277–0.339, P < 0.001). There were 34.8%, 20.6%, and 12.7% of patients with one, two, and three positive LNs who had RS testing, respectively (P < 0.001).

**Table 2:** Predictors of 21-gene testing.

Variables	OR	95% CI	P
Age (years)			
<50	1		
50-64	1.452	1.362-1.548	<0.001
≥65	1.172	1.097-1.252	<0.001
Race/ethnicity			
Non-Hispanic white	1		
Non-Hispanic Black	0.840	0.771-0.915	<0.001
Hispanic (all races)	0.774	0.714-0.837	<0.001
Other			
Histology			
Invasive ductal carcinoma	1		
Invasive lobular carcinoma	1.075	0.995-1.161	0.066
Other	0.770	0.644-0.921	0.004
Grade			
Well differentiated	1		
Moderately differentiated	0.867	0.819-0.919	<0.001
Poorly/undifferentiated	0.522	0.483-0.563	<0.001
T stage			
T1	1		
T2	0.648	0.616-0.681	<0.001
Number of positive lymph nodes			
1	1		
2	0.514	0.484-0.546	<0.001
3	0.307	0.277-0.339	<0.001
PR status			
Negative	1		
Positive	1.466	1.344-1.600	<0.001

### Predictive factors associated with the receipt of chemotherapy

In our study, the proportion of patients who did not undergo 21-gene testing who received chemotherapy was 64.1% and the proportion of patients who received 21-gene testing was 30.8%. Two multivariate logistic regression models were used to determine the predictive factors related to the use of chemotherapy (**Table 3**). The first model included patients without 21-gene testing, and the results showed that younger age, non-Hispanic white, other histological subtypes, higher tumor grade, T2 stage, a higher number of positive LNs, and PR negative diseases were the independent predictive factors associated with the use of chemotherapy (all P < 0.05). Patients aged <50 years had the highest chance of chemotherapy receipt, with an OR of 11.285 compared with those aged ≥65 years (95% confidence interval [CI] 11.359–12.294, P < 0.001). Patients aged 50–64 years were also more likely to receive chemotherapy compared with those aged ≥65 years (OR 6.038, 95% CI 5.653–6.450, P < 0.001). There were 86.2%, 76.5%, and 35.6% of patients aged <50, 50–64, and ≥65 years receiving chemotherapy, respectively (P < 0.001).

**Table 3:** Predictors of chemotherapy receipt in those with and without 21-gene testing.

Variables	No 21-gene testing			21-gene testing		
	OR	95% CI	P	OR	95% CI	P
Age (years)						
<50	1			1		
50-64	0.535	0.490-0.584	<0.001	0.553	0.491-0.623	<0.001
≥65	0.089	0.081-0.097	<0.001	0.207	0.181-0.237	<0.001
Race/ethnicity						
Non-Hispanic white	1			1		
Non-Hispanic Black	0.999	0.902-1.106	0.984	0.962	0.810-1.144	0.663
Hispanic (all races)	0.81	0.738-0.890	<0.001	1.022	0.870-1.199	0.794
Other	0.913	0.820-1.106	0.096	0.84	0.708-0.997	0.840
Histology						
Invasive ductal carcinoma	1			1		
Invasive lobular carcinoma	0.962	0.874-1.060	0.433	0.917	0.787-1.070	0.270
Other	0.766	0.626-0.937	0.009	0.955	0.655-1.393	0.812
Grade						
Well differentiated	1			1		
Moderately differentiated	1.348	1.248-1.455	<0.001	1.429	1.270-1.607	<0.001
Poorly/undifferentiated	2.003	1.827-2.196	<0.001	1.974	1.687-2.311	<0.001
T stage						
T1	1			1		
T2	1.261	1.187-1.341	<0.001	1.224	1.108-1.352	<0.001
Number of positive lymph nodes						
1	1			1		
2	1.613	1.503-1.730	<0.001	1.692	1.495-1.916	<0.001
3	1.889	1.716-2.080	<0.001	2.611	2.109-3.232	<0.001
PR status						
Negative	1			1		
Positive	0.817	0.743-0.899	<0.001	0.808	0.676-0.965	0.019
RS categories						
Low	—			1		
Intermediate	—	—	—	3.821	3.454-4.228	<0.001
High	—	—	—	13.846	10.992-17.441	<0.001

—, indicates none available.

**Figure 3:**
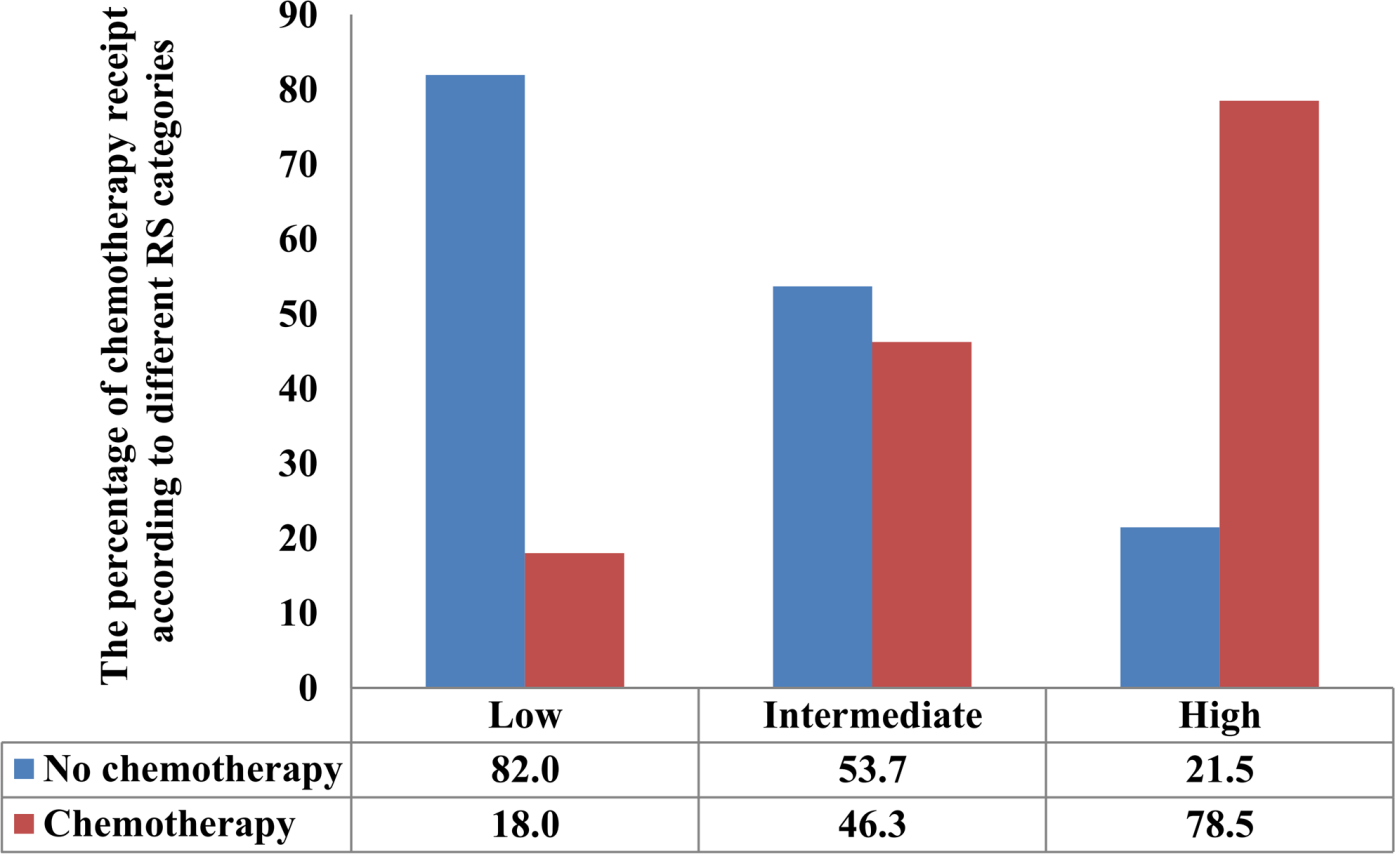
The percentage of receipt of chemotherapy according to different recurrence score categories.

The second model included patients with 21-gene testing; the results showed that younger age, higher tumor grade, T2 stage, a higher number of positive LNs, PR negative, and higher RS were the independent predictive factors associated with the use of chemotherapy (all P < 0.05). Patients with high RS had the highest chance of chemotherapy receipt, with an OR of 13.846 compared with those with low RS (95% CI 10.992–17.441, P < 0.001). Patients with intermediate RS were also more likely to receive chemotherapy compared with those with low RS (OR 3.821, 95% CI 3.454–4.228, P < 0.001). There were 18.0% (n = 1,130), 46.3% (n = 1,576), and 78.5% (n = 453) of patients with low, intermediate, and high RS receiving chemotherapy, respectively (P < 0.001) (**Figure 3**).

### Prognostic effect of 21-gene testing on survival outcomes

**Figure 4:**
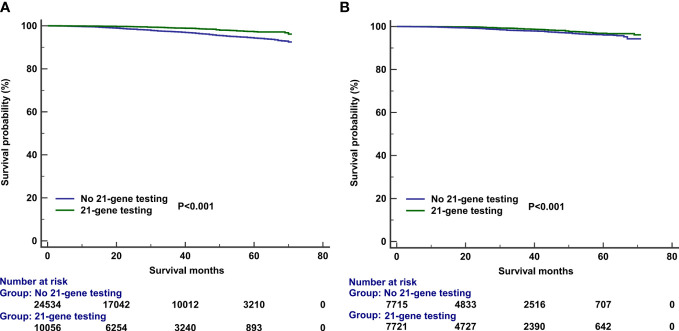
Kaplan–Meier curves of association of 21-gene testing with breast cancer-specific survival before **(A)** and after **(B)** propensity score matching.

**Figure 5:**
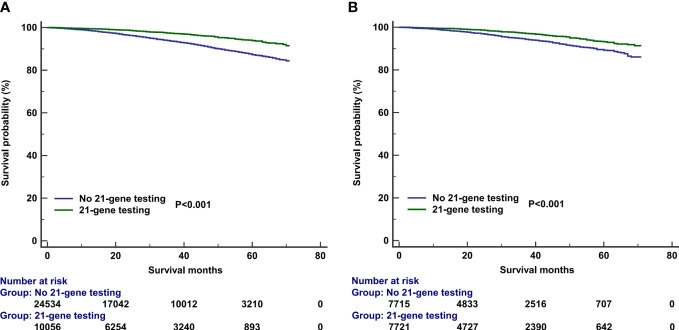
Kaplan–Meier curves of association of 21-gene testing with overall survival before **(A)** and after **(B)** propensity score matching.

The median follow-up of this study was 32 months (range, 0–71 months). A total of 1,938 deaths occurred, including 811 patients who died from BC. Overall, the survival outcomes were excellent in the entire cohort. The 3-year BCSS and OS were 95.0% and 97.8%, respectively. The multivariate prognostic analysis showed that patients who received 21-gene testing had better BCSS (hazard ratio [HR] 0.506, 95% CI 0.406–0.632, P < 0.001) and OS (HR 0.602, 95% CI 0.457–0.793, P < 0.001) than those without 21-gene testing (**Tables 4**, **5**). The 3-year BCSS was 99.2% and 97.4% in those with and without 21-gene testing, respectively (P < 0.001) (**Figure 4A**). The 3-year OS was 97.3% and 93.6% in those with and without 21-gene testing, respectively (P < 0.001) (**Figure 5A**).

**Table 4:** Multivariate prognostic analysis for breast cancer-specific survival before and after propensity score matching.

Variables	Before PSM			After PSM		
	HR	95% CI	P	HR	95% CI	P
Age (years)						
<50	1			1		
50-64	1.155	0.949-1.407	0.151	1.396	0.906-2.152	0.131
≥65	1.638	1.339-2.004	<0.001	1.864	1.223-2.842	0.004
Race/ethnicity						
Non-Hispanic white	1			1		
Non-Hispanic Black	1.257	1.027-1.539	0.026	1.317	0.842-2.060	0.227
Hispanic (all races)	0.98	0.782-1.229	0.864	1.507	0.639-1.747	0.83
Other	0.63	0.464-0.855	0.003	0.785	0.444-1.388	0.406
Histology						
Invasive ductal carcinoma	1			1		
Invasive lobular carcinoma	1.032	0.813-1.308	0.797	0.774	0.465-1.287	0.323
Other	1.021	0.647-1.613	0.928	0.984	0.313-3.093	0.978
Grade						
Well differentiated	1			1		
Moderately differentiated	1.385	1.086-1.765	0.009	1.720	1.118-2.647	0.014
Poorly/undifferentiated	3.279	2.567-4.189	<0.001	4.174	2.662-6.546	<0.001
T stage						
T1	1			1		
T2	2.158	1.850-2.517	<0.001	2.185	1.657-2.882	<0.001
Number of positive lymph nodes						
1	1			1		
2	1.102	0.937-1.297	0.24	1.320	0.950-1.836	0.099
3	1.282	1.049-1.568	0.015	1.510	0.894-2.549	0.123
PR status						
Negative	1			1		
Positive	0.468	0.398-0.550	<0.001	0.617	0.414-0.921	0.018
Surgery procedure						
Breast-conserving surgery				1		
Mastectomy	1			0.933	0.665-1.310	0.689
Radiotherapy	0.941	0.803-1.103	0.455			
No/unknown	1			1		
Yes	0.684	0.592-0.790	<0.001	0.657	0.501-0.863	0.003
Chemotherapy						
No	1			1		
Yes	0.676	0.576-0.795	<0.001	0.863	0.629-1.183	0.359
21-gene testing						
No	1			1		
Yes	0.506	0.406-0.632	<0.001	0.602	0.457-0.793	<0.001

**Table 5:** Multivariate prognostic analysis for overall survival before and after propensity score matching.

Variables	Before PSM			After PSM		
	HR	95% CI	P	HR	95% CI	P
Age (years)						
<50	1			1		
50-64	1.506	1.280-1.772	<0.001	1.758	1.235-2.504	0.002
≥65	3.254	2.783-3.805	<0.001	3.782	2.679-5.341	<0.001
Race/ethnicity						
Non-Hispanic white	1			1		
Non-Hispanic Black	1.263	1.103-1.447	0.001	1.442	1.105-1.882	0.007
Hispanic (all races)	0.901	0.771-1.053	0.19	0.912	0.656-1.267	0.582
Other	0.614	0.498-0.756	<0.001	0.839	0.585-1.203	0.339
Histology						
Invasive ductal carcinoma	1			1		
Invasive lobular carcinoma	0.930	0.801-1.079	0.336	0.797	0.599-1.062	0.121
Other	1.111	0.840-1.470	0.459	0.777	0.367-1.643	0.509
Grade						
Well differentiated	1			1		
Moderately differentiated	1.232	1.081-1.404	0.002	1.355	1.091-1.681	0.006
Poorly/undifferentiated	2.012	1.748-2.317	<0.001	2.038	1.576-2.636	<0.001
T stage						
T1	1			1		
T2	1.625	1.479-1.786	<0.001	1.567	1.327-1.850	<0.001
Number of positive lymph nodes						
1	1			1		
2	1.062	0.955-1.181	0.266	1.254	1.023-1.536	0.029
3	1.218	1.062-1.398	0.005	1.465	1.043-2.058	0.028
PR status						
Negative	1			1		
Positive	0.651	0.579-0.732	<0.001	0.651	0.502-0.844	0.001
Surgery procedure						
Breast-conserving surgery	1			1		
Mastectomy	0.931	0.838-1.035	0.184	0.936	0.755-1.160	0.545
Radiotherapy						
No/unknown	1			1		
Yes	0.632	0.575-0.694	<0.001	0.651	0.551-0.769	<0.001
Chemotherapy						
No	1			1		
Yes	0.502	0.452-0.558	<0.001	0.637	0.514-0.789	<0.001
21-gene testing						
No	1			1		
Yes	0.457	0.399-0.524	<0.001	0.539	0.455-0.640	<0.001

To reduce potential selection bias, a PSM was conducted to balance the patients’ clinicopathological and therapeutic characteristics including the following variables: age, race, tumor grade, histology, T stage, number of positive LNs, PR status, surgical procedure, radiotherapy, and chemotherapy. A total of 7,878 pairs of patients were completely matched. Patients who received 21-gene testing also had better BCSS (HR 0.602, 95% CI 0.457–0.793, P < 0.001) and OS (HR 0.539, 95% CI 0.455–0.640, P < 0.001) compared with those without 21-gene testing (**Tables 4**, **5**). Survival curves are listed in **Figures 4B**, **5B**.

### Prognostic effect of 21-gene results on survival outcomes

**Figure 6:**
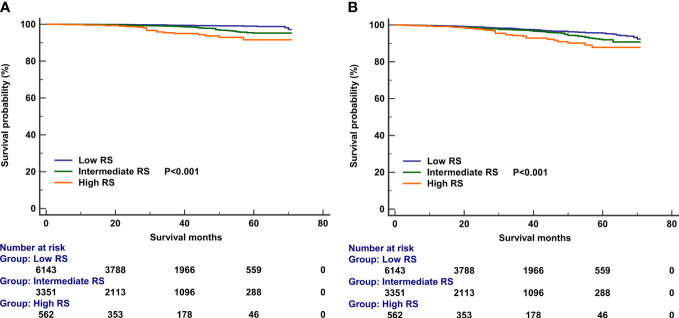
Kaplan–Meier curves of association of recurrence score categories with breast cancer-specific survival **(A)** and overall survival **(B)**.

In those with 21-gene testing (n = 10,247), the multivariate prognostic analysis indicated that the RS was the independent prognostic factor associated with survival outcomes. Those with high RS had significantly lower BCSS (HR 4.158, 95% CI 2.147–8.082, P < 0.001) and OS (HR 2.079, 95% CI 1.353–3.195, P = 0.001) compared with those with low RS. In addition, patients with intermediate RS also had significantly lower BCSS (HR 2.586, 95% CI 1.604–4.169, P < 0.001) and OS (HR 1.411, 95% CI 1.085–1.836, P = 0.010) compared with those with low RS (**Table 6**). The survival curves according to the RS cohorts are listed in **Figure 6**.

**Table 6:** Multivariate prognostic analysis for breast cancer-specific survival and overall survival in those with 21-gene testing.

Variables	BCSS			OS		
	HR	95% CI	P	HR	95% CI	P
Age						
<50	1			1		
50-64	2.033	0.952-4.342	0.067	2.491	1.466-4.234	0.001
≥65	3.032	1.425-6.448	0.004	4.777	2.842-8.030	<0.001
Race/ethnicity						
Non-Hispanic white	1			1		
Non-Hispanic Black	1.545	0.850-2.809	0.154	1.499	1.025-2.192	0.037
Hispanic (all races)	1.055	0.506-2.198	0.887	1.086	0.698-1.689	0.716
Other	0.446	0.140-1.419	0.171	0.531	0.272-1.037	0.064
Histology						
Invasive ductal carcinoma	1			1		
Invasive lobular carcinoma	1.195	0.637-2.242	0.579	0.813	0.540-1.223	0.32
Other	0.685	0.095-4.952	0.708	1.026	0.421-2.501	0.954
Grade						
Well differentiated	1			1		
Moderately differentiated	1.668	0.883-3.151	0.115	1.315	0.830-1.552	0.427
Poorly/undifferentiated	0.792	1.385-5.629	0.004	1.419	0.952-2.115	0.085
T stage						
T1	1			1		
T2	1.656	1.103-2.488	0.015	1.497	1.169-1.918	0.001
Number of positive lymph nodes						
1	1			1		
2	1.206	0.714-2.036	0.483	1.108	0.801-1.533	0.535
3	1.814	0.892-3.689	0.1	1.515	0.938-2.447	0.089
PR status						
Negative	1			1		
Positive	0.596	0.348-1.021	0.059	0.720	0.493-1.050	0.088
Surgery procedure						
Breast-conserving surgery	1			1		
Mastectomy	0.755	0.454-1.256	0.279	0.920	0.669-1.265	0.608
Radiotherapy						
No/unknown	1			1		
Yes	0.649	0.395-1.065	0.087	0.707	.552-0.904	0.006
Chemotherapy						
No	1			1		
Yes	1.079	0.682-1.709	0.745	0.830	0.613-1.124	0.228
RS categories						
Low	1			1		
Intermediate	2.586	1.604-4.169	<0.001	1.411	1.085-1.836	0.010
High	4.158	2.147-8.052	<0.001	2.079	1.353-3.195	0.001

## Discussion

In the current study, a large cohort from the SEER Oncotype DX Database was used to investigate the practice patterns and the potential benefit of 21-gene testing in BC patients with ER+/HER2− and one to three positive LNs. Our results showed that adjuvant chemotherapy recommendations for this population mainly relied on genomic profiling assays in the current era. Moreover, the performance of 21-gene testing was associated with improved survival outcomes. Our findings support the routine use of RS testing in this population.

With the rapid progress in the understanding of BC biology, the current treatment of BC is mainly based on the results of genomic profiling assays. As the most widely used genomic profiling assay, 21-gene RS is the most important factor influencing the treatment strategy of N0 early-stage BC. The NCCN guidelines recommend the use of the 21-gene expression assay in the decision-making of both N0 and N+ (one to three LNs) ER+ BC (4). In contrast, the ASCO guidelines state that the clinician should not use the 21-gene expression assay to guide adjuvant chemotherapy decisions in N+ and ER+/HER2− BC patients (9). In a National Cancer Database study including 72,897 patients with N+ BC diagnosed between 2010 and 2013, the receipt of 21-gene testing was 15% in 2013 and 24% in 2013 (17). In our study, there were 21.2% of patients who had 21-gene testing in 2010, which was significantly increased to 36.8% in 2015 (P < 0.001). However, the recommendation of 21-gene testing was also lower than those with N0 disease, with 21.8% having 21-gene testing in the years 2004–2011, and nearly half of patients had 21-gene testing in 2013–2015 in real-world studies (18, 19). This may reflect the practice pattern of most clinicians prescribing chemotherapy to most patients with one to three LNs even with estrogen sensitivity.

The distribution of RS in N0 BC was 48.8%–53.7%, 38.9%–40.7%, and 7.4%–10.4% in patients who had low, intermediate, and high RS, respectively (20, 21). In our study, there were 61.1%, 33.2%, and 5.6% of patients who had low, intermediate, and high RS, respectively. Similar findings were observed in the other studies that included patients from Japan (22), Canada (23), and Israel (24), which suggested that the RS also had a similar distribution in most populations. However, a study from China showed that there were 21.4%, 53.1%, and 25.5% of patients who had low, intermediate, and high RS in those with N1 disease, respectively (11). Only 98 patients who had N1 diseases limited the study to the general population. In our previous study, we found similar distribution, chemotherapy use, and prognostic prediction of the 21-gene RS between Chinese and white American BC patients (25). For Chinese female patients, approximately one-quarter suffered from N1 disease at the time of BC diagnosis (26). Therefore, 21-gene testing could also play an important role in treatment decision-making for the Chinese population with a rapidly growing incidence rate of BC (27).

A review including a series of the study showed that the use of 21-gene testing had an 18%–69% reduction in chemotherapy recommendation in patients with N+ BC (28). In a study by Stemmer et al., they found that 21-gene testing was significantly associated with lower odds of receiving chemotherapy (OR 0.16, P < 0.001) (24). They also found that in those with 21-gene testing, 7.1%, 37.0%, and 100% of patients with low, intermediate, and high RS received chemotherapy, respectively. In our study, the proportion of chemotherapy use in patients without 21-gene testing was 64.1% and 30.8% in those receiving 21-gene testing. In our study, 18.0%, 46.3%, and 78.5% of low-, intermediate-, and high-RS patients received chemotherapy. Our findings demonstrated that the recommended rate for adjuvant chemotherapy was significantly lower in patients undergoing 21-gene testing. For those with N0 disease, the rates of chemotherapy use were 1.4%, 23.7%, and 87.2% in low-, intermediate-, and high-RS patients, respectively (20). There were large differences in the probability of each subgroup receiving chemotherapy after 21-gene testing for patients with N1 and N0 stages, especially for patients with low and intermediate RSs. The above results suggest that although genetic testing technology can better predict the value of being chemotherapy-free in N0 patients, it is still doubtful whether N1 patients can safely avoid chemotherapy.

In our study, there were 29.2% of patients who had 21-gene testing and patients with favorable prognostic factors were more likely to receive 21-gene testing. Moreover, those with three positive LNs had the lowest chance of 21-gene testing. There were 34.8%, 20.6%, and 12.7% of patients with one-, two-, and three-LN metastases who had 21-gene testing, respectively. Similar results were found from the study by Roberts et al., which included 30,410 patients with N+ BC, and the receipt of 21-gene testing was low in patients with high-risk factors (29). Our findings, along with the above study, showed that clinicians may choose not to perform 21-gene testing in more advanced patients with historical indications for chemotherapy.

In the current NCCN guideline, the recommendation of treatment for one to three positive LNs with ER+/HER+- is based on the results of 21-gene testing. In those without 21-gene testing, chemotherapy and endocrine therapy or endocrine therapy alone are recommended, whereas endocrine therapy is recommended in those with RS <26 and chemotherapy and endocrine therapy is recommended for those with RS ≥26 (5). Specifically, age was used as a main predictive factor for chemotherapy recommendation in our study in patients without 21-gene testing; there were 86.2%, 76.5%, and 35.6% of patients aged <50, 50–64, and ≥65 years receiving chemotherapy, respectively (P < 0.001). In those with 21-gene testing, RS was the main predictive factor for chemotherapy in patients; there were 8.0%, 46.3%, and 78.5% of patients with low, intermediate, and high RS receiving chemotherapy, respectively. Several studies showed that in those with one to three positive LNs, the overall change rate of treatment was 49%–55.1%, 27%–59.3%, and 0%–18% in those with low, intermediate, and high RSs, respectively (22, 30). Although the overall change rate of treatment was unavailable in the retrospective analysis, our results showed that there were 61.4% of patients who received chemotherapy in the no 21-gene testing cohort, which was significantly higher than in the patients with 21-gene testing (30.8%) (P < 0.001). This is remarkable in that the decision to omit chemotherapy in those with one to three positive LNs according to the 21-gene testing is similar to those with N0 disease. Therefore, tumor biology with genomic assays has been widely used in treatment-decision making of BC, which has beyond the traditional clinical and pathological characteristics in the selection of adjuvant treatment.

In patients with N0 disease, numerous studies have confirmed that RS has an important correlation with patient survival (31, 32). For patients with one to three positive LNs, studies have shown that RS can also affect various survival endpoints, including locoregional control, distant metastasis, and OS (10–13). In our study, we also found that patients with higher RS had worse survival outcomes. Our finding suggests that the RS provides additional prognostic assessment information of tumor biology beyond traditional clinicopathological factors for patients with one to three positive LNs. Given the results of our study and the previous findings, the 21-gene expression assay should be part of routine tests for BC patients with ER+/HER2− and one to three positive LNs.

For clinically high-risk patients, performing 21-gene testing can help clinicians identify subgroups of patients with favorable prognoses who can be safely spared from adjuvant chemotherapy.

To our knowledge, no studies have focused on the effect of 21-gene testing on the survival of T1-2N1 patients. In a previous study by Pomponio et al., they included patients with N0 and tumor size ≤1 cm and found that patients receiving 21-gene testing had better survival compared with those without 21-gene testing (33). Overall, the survival outcomes in our cohort were excellent regardless of the 21-gene testing. The 3-year BC related-death rates were 0.8% and 2.6% in those with and without 21-gene testing, respectively (P < 0.001). Our multivariate analyses showed that the use of RS testing was associated with better survival outcomes after adjustment of known prognostic variables. Similar results were found using PSM. A previous study included patients with N0 disease, which found that 21-gene testing is cost-effective for patients with intermediate- and high-RS cohorts, but not for the low-RS cohort (34). In patients with one to three positive LNs, the use of the 21-gene expression assay could be also a cost-effective strategy in this population (23).

Several limitations should be acknowledged in our study. First, the data of our study were from a retrospective database, which was inherently biased. Second, we were unable to observe clinician influence on patient treatment decisions, only practice patterns. Third, since outcome data regarding locoregional recurrence and distant metastasis are not available in the SEER database, and the median follow-up was only 32 months, the impact of adjuvant chemotherapy on survival outcomes according to different RS categories was not assessed. However, the primary strength of this study was that we used a population-based cohort to investigate the role of 21-gene RS testing in patients with one to three positive LNs. Our study could contribute to the understanding of the role of 21-gene RS testing in chemotherapy decision-making and prognostic prediction in this population.

## Conclusions

In conclusion, our study suggests that the 21-gene expression assay is frequently and increasingly used for treatment decision-making in ER+/HER2- BC patients with one to three positive LNs and its use is related to lower rates of adjuvant chemotherapy. Performance of 21-gene testing in BC with one to three positive LNs is associated with better survival outcomes. Our study supports the routine use of 21-gene testing in the clinical practice of this population.

## Data availability statement

The raw data supporting the conclusions of this article will be made available by the authors, without undue reservation.

## Ethics statement

Ethical review and approval was not required for the study on human participants in accordance with the local legislation and institutional requirements. Written informed consent for participation was not required for this study in accordance with the national legislation and the institutional requirements.

## Author contributions

G-QL and S-JX drafted the manuscript. S-GW acquired the datasets. S-GW and Z-YH conceived the study. S-GW conducted the statistical analyses. G-QL, S-JX, and Z-YH participated in the study design. All authors read and approved the final manuscript.
